# Does promising facilitate children’s delay of gratification in interdependent contexts?

**DOI:** 10.1098/rsos.250392

**Published:** 2025-05-07

**Authors:** Rebecca Koomen, Owen Waddington, Leonor Santana Miranda Goncalves, Bahar Köymen, Keith Jensen

**Affiliations:** ^1^ School of Collective Intelligence, Mohammed VI Polytechnic University, Ben Guerir, Morocco; ^2^ Division of Psychology, Communication and Human Neuroscience, The University of Manchester, Manchester, UK

**Keywords:** delay of gratification, cooperation, marshmallow paradigm, promises

## Abstract

For cooperation to succeed, individuals must often ‘delay gratification’ and forego an immediate reward for a larger delayed reward that is co-produced through the cooperative act. This experiment asked whether a promise to wait increased children’s propensity to coordinate with their partner by waiting to eat their own treat. In this first cooperative marshmallow test conducted online, 5- to 6-year-old UK-based children (*n* = 66) interacted from their homes via video call with a confederate child who either promised not to eat his treat (promise condition) or expressed the possibility that he might eat his treat (social risk condition). Across the full dataset and a reduced dataset in which participants were not accidentally interrupted during the task (*n* = 48), children in the promise condition waited longer to eat their treat than children in the social risk condition. Younger children, but not older children, also successfully delayed gratification more often in the promise condition than in the social risk condition. Thus, even when communication is one-sided in an interdependent marshmallow task, explicit promises can support children’s motivation to delay gratification relative to explicit uncertainty.

## 1. Introduction

Humans are an exceptionally cooperative species. Our cooperative skills facilitate frequent and peaceful interaction with strangers, the co-construction of culture and technology, as well as countless scientific and artistic endeavours. This so-called hyper-cooperativeness or ultrasociality [1,2] is made possible by a suite of social cognitive abilities and motivations. In cases of social interdependence, in which partners are mutually dependent upon one another, cooperation requires behavioural coordination, but also frequently delay of gratification (DoG). For example, in order for two colleagues to write a joint report, they must coordinate their efforts over time, during which they must also avoid short-term temptations such as social media, watching a movie, etc. They must both self-maintain these behaviours—coordination and delay of gratification—in order to reap the professional rewards associated with their cooperation.

Social interdependence is thought to have played a central role in the evolution of human cooperative skills and motivations [2–4]. Psychologically, interdependence can be structured by joint intentions [5], commitments and a sense of obligation to one’s partner(s) [6]. Indeed, German 3-year-old children will invest in a joint endeavour after making an explicit commitment to do so, but by the age of 5 years children mutually recognize the nature of their interdependence in the same joint endeavour in order to cooperate without any explicit commitments [7]. This demonstrates that, even from a young age, social interdependence can generate a sense of obligation between partners.

When interdependence requires delay of gratification, this sense of obligation can also lead to increased effort, and therefore increased cooperative success. This has been shown experimentally in children using a modification to the traditional marshmallow test paradigm [8]. In this study, the outcomes of two children were linked such that neither was rewarded with a larger, delayed reward unless they both waited to eat their initial reward (i.e. treat). Across two disparate cultures (Germany and Kenya), 5- and 6-year-old children waited more reliably to eat their treat when they were interdependent with a partner than when they experienced a traditional, independent marshmallow test. This result was found in the absence of explicit commitments between partners; participants were given the task after being separated into two rooms and could not, therefore, communicate about it.

Even in the absence of interdependence, a child’s social context can impact their readiness to delay gratification. For example, knowledge that one’s ingroup prefers DoG [9], and knowledge that a peer or teacher will learn of one’s DoG performance [10] both increase American and Chinese children’s DoG likelihood. Beyond reputation management, rational calculations about the likelihood of later receiving a larger reward are also influenced by children’s social experiences. If children trust the experimenter [11], if they hold a generalized trust for others [12] or if they find the environment to be generally reliable [13], children (USA and China) are more likely to delay gratification. One way to signal social reliability in an interdependent context is to promise.

Promises are a normatively binding form of explicit commitment that can powerfully influence children’s behaviour and bolster cooperative potential. Operationally defined, a promise is a self-referential speech act with which a speaker signals commitment and intention to some future action [14]. A child who has been promised something will expect their partner to follow through with the promised act, which sets promises apart from other speech acts directed at future actions such as predictions or assertions [15]. Similarly, when a 5-year-old child makes a promise to an adult, they are much more likely to subsequently adhere to their promise (China [16]). American 5-year-olds also tell the truth more readily after promising to do so [17,18]. The effect of promises extends beyond individual prosociality into interdependent cooperation. Kanngiesser *et al*. [19] tested children’s behaviour in a joint cleaning task with an experimenter. When German 3- and 5-year-olds promised to continue in the task, they resisted the temptation to play with a more exciting toy and cleaned for longer than children who did not promise [20]. Moreover, 3- and 5-year-olds (USA and Germany) verbally objected to a partner breaking their promise, citing promise norms as justification for their objection [21–23]. These findings indicate that young children experience a normative sense of obligation to uphold their promises and expect others to do the same.

An important question is whether promises inspire enough trust in a partner to delay gratification, particularly when contrasted with the social risk of a potentially untrustworthy partner. From a developmental perspective, when an interdependent task requires delay of gratification in order to achieve joint success, can promises elicit greater effort and heightened commitment in children? A further question is whether the marshmallow test paradigm can be adapted to an online audience. Recent evidence suggests that moderated online studies yield mixed results in comparison with laboratory-based experiments; some yield similar results [24] whereas others do not [25]. Given face-to-face testing restrictions during the COVID-19 pandemic, this, to our knowledge, represents the first cooperative marshmallow study to be conducted online thereby adding to the growing body of literature concerning the validity of remote testing methods.

The current study followed the interdependent condition in the Koomen *et al*. [8] paradigm with 5- and 6-year-old children. This study tested UK-based children in a joint marshmallow test with two interdependent conditions. We compared children’s DoG performance when interacting with a confederate who either promised not to eat his treat (promise condition) or who expressed the possibility that he might eat his treat (social risk condition). Children’s outcomes were ostensibly interdependent with the confederate’s because if either child failed to delay gratification, neither would get a second reward. DoG performance was measured in two ways: whether or not children successfully delayed gratification (i.e. did or did not consume their treat) and the duration of delayed gratification (i.e. latency to nibble, eat or lick, up to a maximum of 10 min). We predicted that children would delay gratification more often (i.e. not consume their treat) in the promise condition than in the social risk condition. We further predicted that children would wait longer before consuming their treat in the promise condition than in the social risk condition.

## 2. Method

The procedure, hypotheses, sample size, exclusion criteria and statistical analyses were pre-registered (https://osf.io/ze9jb). Any deviation from the pre-registration is outlined below.

### 2.1. Participants

Forty-eight 5- to 6-year-olds (*M*_age_ = 5.96, s.d. = 0.54, 25 girls) initially participated in the study and were randomly assigned to one of two conditions. Five additional children were excluded owing to refusal to play the game (*n* = 3), serious technical issues (one) and experimenter error (one). Children who were native speakers of English were recruited from a database of families living in northwest England. We did not collect individual data about children’s socioeconomic or ethnic backgrounds, but families in this database come from predominantly White, middle-class backgrounds. Data collection first took place between May 2021 and February 2022. Parental consent (written and verbal) and child assent (verbal) were obtained before participation in the study.

To control for any effects of unavoidable (accidental) distractions in children’s home environments during participation, an exclusion ‘stoplight’ system was designed in which children were excluded and time was added to trials, depending on the predicted severity of the distraction (see the electronic supplementary material, A for a description of the stoplight events). To estimate the duration of distractions, a stopwatch was used in-trial by the experimenter. The beginning and end of the event (e.g. frozen webcam) was timed and the trial was extended accordingly. This was to ensure all children were observed under the same experimental conditions for the full length of the study (10 min). Using this stoplight system, the study consisted of a full dataset which contained all participants and a reduced dataset which excluded participants for whom the stoplight system deemed their distractions to have been significant. By removing all instances of distraction, the reduced dataset most closely mimicked the controlled conditions of a laboratory setting in which the marshmallow paradigm is normally tested.

In total, 17 children from the original sample experienced distractions or minor technical issues. These distractions were not believed to be marked enough to warrant total exclusion from the study but were sufficient to discount the child from the reduced dataset. Distractions included: minor internet/IT disruption (*n* = 10), interruption from parents, siblings or pets (four) or technology, such as notifications (three). To ensure that the reduced dataset met our preregistered sample size, 18 additional children were tested (data collection: November 2024–January 2025), with one further child removed from the reduced dataset owing to parental interference. Thus, a sum of 66 children aged 5- to 6-years-old (*M*_age_ = 5.96, s.d. = 0.55, 37 girls) comprised the full dataset, with 48 of them (*M*_age_ = 5.98, s.d. = 0.58, 28 girls) featured in the reduced dataset: 24 in the promise condition, and 24 in the social risk condition.

### 2.2. Materials

Children were shown two pre-recorded videos of a confederate child who either promised not to eat his treat or expressed the possibility that he might eat his treat. Videos were presented in PowerPoint (six slides; see the electronic supplementary material, B).

### 2.3. Procedure

The study protocol was approved by the Research Ethics Committee of The University of Manchester (ref: 2021-11045-17831). Children and their parents joined a Zoom meeting which was divided into two phases: the set-up phase (with parents only) and the experimental phase (with the child only). During the set-up phase, parents were asked by the experimenter (E) to prepare a ‘food confectionary’ of their choosing and to find a quiet room with minimal potential distractions (see the electronic supplementary material, C for instructions emailed to parents). Parents typically selected treats that were among their child’s favourites. All treats were made visible (i.e. unwrapped) to ensure children had the same access to the treat. The recording device (e.g. webcam) was also adjusted where necessary to ensure that the child and treat remained visible throughout the test session. Once the set-up was complete, parents were kindly asked to bring their child into the room and then exit quietly.

In the experimental phase, E screen-shared a PowerPoint presentation with the child. E explained that another child (the confederate), who was already familiar with the rules of the game, would soon be joining them. E proceeded to explain the rules to the participant child. Participants were informed that both they and the confederate child had a treat in front of them, which they could eat at any point. E then noted that she would be stepping outside to attend to some work for an undisclosed period of time and if both the participant child’s and the confederate child’s treat remained once E returned, both would receive another treat. However, if either ate their treat prior to E’s return, then neither would receive a second treat. Children would also forgo their second treat if either left their seat or spoke to anyone before E returned. Thus, participants were led to believe that they would only get another treat if both they and the confederate child waited to eat their respective treats. Comprehension checks followed and feedback was given (see the electronic supplementary material, B for the full script).

Participants were then presented with a pre-recorded video of the confederate child sitting with a cookie in front of him. To discourage the participant child from attempting to interact with the confederate, E stated that, owing to technical problems with Zoom, the confederate child could neither see nor hear them (though the participant child could see and hear the confederate). Throughout, E spoke to the confederate child as though it was a live interaction to convince the participant child that they were playing with a genuine peer. Before the activity began, E asked both children whether they were ready to play and the confederate issued a brief statement. In the promise condition, the confederate child promised not to eat his treat (‘I promise I won’t eat this cookie’). In the social risk condition, the confederate child expressed the possibility that he might eat his treat (‘I think I will eat this cookie’). For young children, ‘I think’ expresses uncertainty or hedging (e.g. ‘maybe’) as they are unlikely to refer to mental states [26,27]. In this way, we compare the promise condition with one in which the confederate states uncertainty, making the risk involved explicit. E repeated the confederate’s utterance to the participant child and then left to work off-camera: ‘I’m going now. I won’t be able to see you once I leave. But I’ll see you when I get back’. Importantly, the participant child was unable to see, hear nor interact with the confederate child during the waiting activity. Participants only saw an empty background. After 10 min, E returned and asked whether the participant child had consumed (ate/licked/nibbled) their treat. In another pre-recorded video, E then asked the confederate child whether he had consumed his treat. Across both conditions, the confederate always refrained from eating his treat. To end the study on a positive note, E suggested that both children should get a second treat irrespective of whether or not the participant child had managed to delay gratification.

Sessions were recorded for coding purposes and lasted approximately 15−20 min.

### 2.4. Coding

We coded whether or not children successfully delayed gratification. We then coded for how long (i.e. latency) children delayed their gratification and whether or not they experienced a minor distraction. Latency was calculated (in seconds) from when the child was left unattended to when the child ate/licked/nibbled her treat or broke a rule (e.g. left her chair, initiated conversation with others). Children who successfully delayed gratification and refrained from all of the above received a latency score corresponding to the full trial duration (600 s). In cases where distractions (e.g. frozen webcam) occurred before children consumed their treat or broke a rule, this delay was included and not subtracted from their total wait time. However, these children were then omitted from the reduced (non-distracted) dataset. All coding was done from video recording. Two coders, blind to condition, coded the delay of gratification, latency and distraction data. Interrater reliability checks were conducted on a representative sample totalling 18% of the data (12 children, six per condition). Agreement was high: children’s delay of gratification (*k* = 1); latency (intraclass correlation coefficient [ICC] = 0.997); distraction (*k* = 0.92).

## 3. Results

Parents chose a variety of food items for use in the delay of gratification task. These included: chocolate treats (28), candy (16), biscuits (11), marshmallows (eight), potato chips (one), fruit (one) and nuts (one). Two sets of pre-registered analyses were then conducted: one on whether or not children successfully delayed their gratification, and one on the duration of their delay of gratification (in seconds).

**Figure 1.**
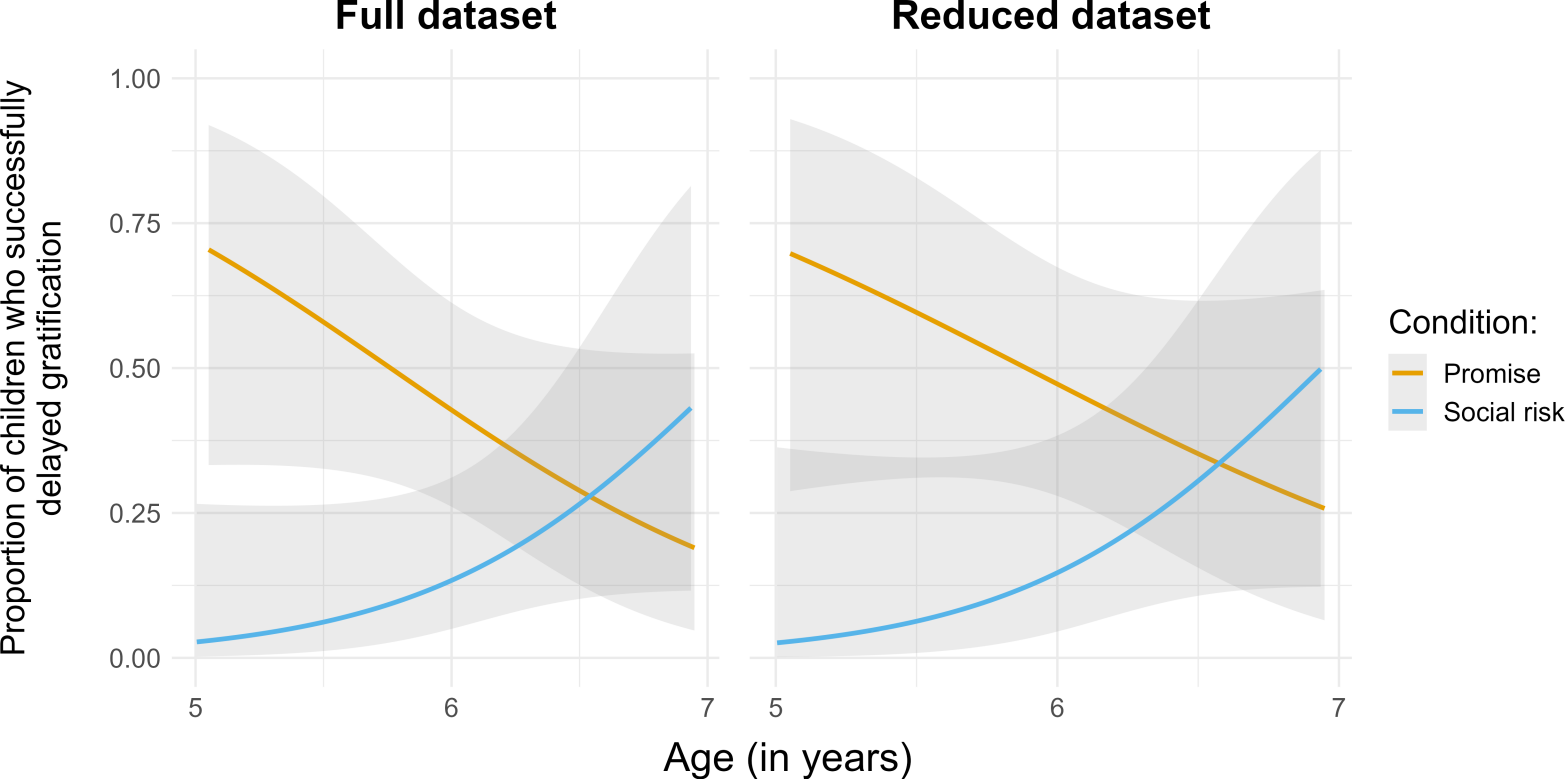
Children’s delay of gratification by age and condition across the full (*n* = 66) and reduced dataset (*n* = 48). Lines represent fitted data with 95% confidence intervals.

First, to identify whether children’s delay of gratification differed by condition, we fitted two generalized linear models with binomial error distribution (one with the reduced dataset which excluded the 18 children who experienced minor distractions, and one with the full dataset which included all 66 children). The response variable was the binary measure of whether or not the child successfully delayed gratification. For the full dataset analysis, the full model included the predictors: age (treated as a continuous variable, *z*-transformed), condition (promise, social risk), their interaction and the control predictors of gender and distraction (distraction, no distraction). The null model included gender and distraction only. The full model improved the fit ($\chi_{3}^{2}=12.86$, *p* = 0.005). The interaction between age and condition was significant ($\chi_{1}^{2}=5.94$, *p* = 0.015). Younger children delayed gratification more often in the promise condition than in the social risk condition. Older children’s delay of gratification did not differ between conditions (see figure 1). The main effect of gender was also significant ($\chi_{1}^{2}=8.19$, *p* = 0.004), with girls successfully delaying gratification (40.5% success rate) more than boys (10.3%). However, as gender was a control variable, we refrain from making any causal interpretations related to this main effect [28].

For the reduced dataset analysis, the full model included the same set of predictors with the exception of the distraction term, which was removed. The null model included gender only. The full model improved the fit ($\chi_{3}^{2}=11.19$, *p* = 0.011). The interaction between age and condition was significant ($\chi_{1}^{2}=4.77$, *p* = 0.029). Without distractions, younger children delayed gratification more often in the promise condition than in the social risk condition. Older children’s delay of gratification did not differ between conditions (see figure 1). The main effect of gender was also significant ($\chi_{1}^{2}=5.84$, *p* = 0.016). Girls successfully delayed gratification (42.9%) more than boys (15%).

**Figure 2.**
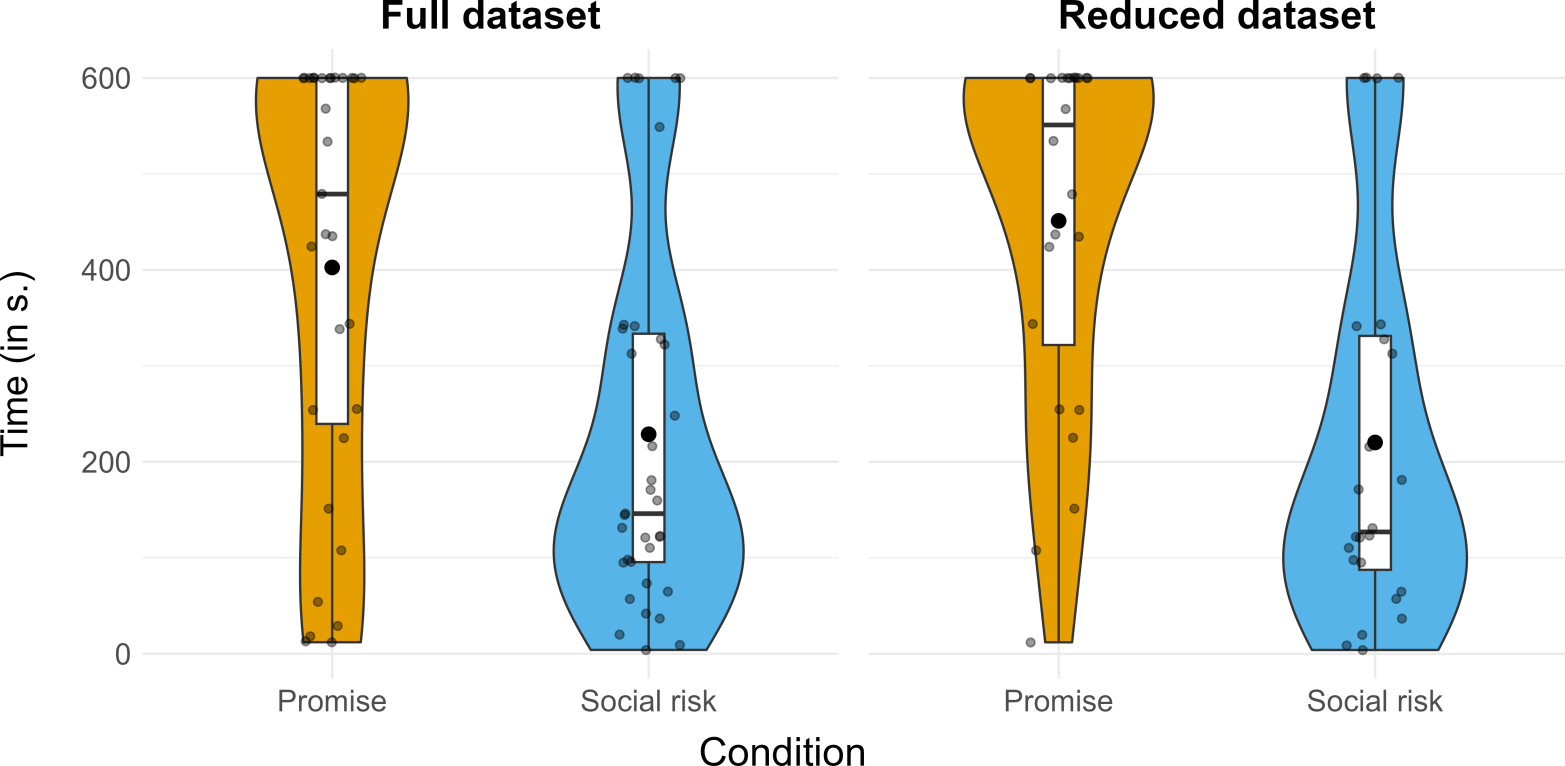
Duration of children’s delay of gratification by condition across the full (*n* = 66) and reduced dataset (*n* = 48). For each grouping, black dots show the individual datapoints. In the boxplots, the bold black lines represent the median and the bold black dots show the mean. Curves represent the probability distribution of the data.

Second, to determine whether the duration of children’s delay of gratification varied by condition, we conducted two between-subjects ANCOVAs (one for each dataset, respectively). Because treating age as a continuous variable violates ANOVA assumptions, this approach represents a departure from our pre-registered analysis. The response variable was the duration of which children delayed their gratification (in seconds). For the full dataset analysis, predictors included: age (treated as a continuous covariate, *z*-transformed) and condition, their interaction, gender and distraction as a binary predictor (distraction, no distraction). The interaction between age and condition was not significant (*F*_1,60_ = 3.36, *p* = 0.072, *ƞ_p_^2^* = 0.053). Only the main effect of condition was significant (*F*_1,60_ = 10.35, *p* = 0.002, *ƞ_p_^2^* = 0.147). Children delayed gratification for longer in the promise condition (*M* = 403, s.d. = 224, 95% confidence intervals (CI) [320 485]) than in the social risk condition (*M* = 229, s.d. = 194, 95% CI [162 295]) despite experiencing various distractions (see figure 2).

For the reduced dataset analysis, the model was the same as the previous ANCOVA except distraction was removed as a predictor. The interaction between age and condition was not significant (*F*_1,43_ = 1.52, *p* = 0.225, *ƞ_p_^2^* = 0.034). The main effects of condition (*F*_1,43_ = 19.15, *p* < 0.001, *ƞ_p_^2^* = 0.308) and gender were significant (*F*_1,43_ = 5.49, *p* = 0.024, *ƞ_p_^2^* = 0.113). Without distractions, children delayed gratification for longer in the promise condition (*M* = 451, s.d. = 187, 95% CI [372 530]) than in the social risk condition (*M* = 220, s.d. = 201, 95% CI [135 305]; see figure 2). Girls delayed gratification for longer (*M* = 382, s.d. = 232, 95% CI [292 472]) than boys (*M* = 271, s.d. = 203, 95% CI [176 366]).

To identify whether children who failed to delay gratification for the full 10 min (600 s) term still waited longer to consume their treat in the promise condition than in the social risk condition, we conducted two additional exploratory between-subjects ANCOVAs (one for each dataset, respectively). The models were the same as the previous ANCOVAs, except participants who had successfully delayed gratification were excluded from the analyses, leaving 48 participants in the full analysis and 33 in the reduced analysis. For the full dataset analysis, no significant interaction (*F*_1,42_ = 0.01, *p* = 0.913, *ƞ_p_^2^* < 0.001) nor main effects were found (*F*s_1,42_ < 2.43, *p*s > 0.127, *ƞ_p_^2^*s < 0.055). For the reduced dataset analysis, the interaction between age and condition was not significant (*F*_1,28_ = 0.77, *p* = 0.388, *ƞ_p_^2^* = 0.027). Only the main effect of condition was significant (*F*_1,28_ = 10.20, *p* = 0.003, *ƞ_p_^2^* = 0.267). Children who failed to delay gratification but experienced no distractions still waited longer in the promise condition (*M* = 325, s.d. = 172, 95% CI [221 429]) than in the social risk condition (*M* = 144, s.d. = 111, 95% CI [92 196]).

## 4. Discussion

Successful cooperation often relies, in part, on individuals maintaining delayed gratification. Here, we demonstrate that 5- to 6-year-old children are better able to delay gratification and can do so for longer when they believe their partner to be a reliable cooperator through the act of promising. These results were found irrespective of whether or not participants experienced minor distractions (e.g. brief interruptions from parents, siblings or pets) during the course of the experiment. Thus, the present study builds upon the Koomen *et al*. [8] interdependence condition in an online testing environment, and goes further to show that when a partner promises to wait for their treat, children’s willingness to delay gratification is greater than when the partner expresses uncertainty.

Though children regardless of age waited longer to eat their treat when the recipient of a promise, younger children were found to successfully delay gratification more often in the promise condition than in the social risk condition. Younger children’s tendency to wait the entire 10 min term in the promise condition might suggest a more naïve understanding of—and trust in—explicit social commitments insofar as they strongly expect promises to be upheld [20]. Alternatively or additionally, the rarity with which they delayed gratification in the social risk condition perhaps suggests that expressions of uncertainty in the context of an implied joint commitment were interpreted as signs of potential defection or an intention to act. In the present study, younger children may thus have been especially convinced by the confederate’s promise to refrain from consuming his treat, and especially concerned when he voiced uncertainty (i.e. ‘I think I will eat this cookie’). This, in turn, increased their motivation to delay gratification in the former and decreased their incentive in the latter. With age, however, children encounter more broken promises and learn that commitments are not always fulfilled (e.g. [22]). Expressions of indecision (e.g. ‘I think...’) may also be increasingly viewed as signs of waver and not necessarily as markers of impending defection [26,27]. Older children might thus view explicit social commitments less as absolutes and, instead, understand them to be malleable to change. As a result, despite waiting longer in the promise condition, the confederate’s utterances appeared to have relatively less of an effect on older children’s delay of gratification. During the late preschool period, children may begin to adopt a more nuanced perspective when evaluating explicit markers of commitment.

The fact that a DoG difference between conditions was found across both datasets suggests that the effect itself is reliable. The distractions experienced by children were, however, found to influence their delay of gratification under certain circumstances. This was most evident among children who failed to delay their gratification for the full term. In the absence of distractions, children who failed to delay gratification nonetheless waited longer to consume their treat when the confederate issued a promise to wait than when he suggested he might not. This was not the case, however, when participants who had experienced minor distractions during the task were included in the analysis. Certain distractions appeared detrimental to children’s delay of gratification. Occasionally, for instance, pets or siblings entered the room causing participants to leave their seats thereby failing the task earlier than they might have done had the distraction not occurred. Other distractions, however, likely enabled children to delay gratification for longer than they would have otherwise (e.g. notifications on their device, playing with nearby stationery or toys). Indeed, positively valenced self-distraction can aid in children’s DoG maintenance [29,30]. For children unable to delay their gratification, then, these minor distractions appeared to null the effect that was present in their absence. Removing data from the dataset that included minor distractions (from a pre-defined stoplight system) therefore aligned the dataset more closely with the conditions of a laboratory-based testing environment, in which distractions can be controlled for all participants equally. Thus, the stoplight system of distractions provides a useful methodological rubric for future marshmallow tests to be conducted remotely.

In addition to social interdependence, our manipulation involved the confederate child either promising to delay gratification or expressing uncertainty about not delaying gratification. In Koomen *et al*. [29], both children faced the implicit risk that their partner would defect and not wait. In the present study’s manipulation, the confederate explicitly stated this same risk by musing about the possibility of eating his treat. A question for future studies is whether an implicit social risk carries less weight than an explicit social risk. One way this could be done is by comparing an explicit form of commitment (or lack thereof) against an implicit risk condition, in which the confederate makes no reference to the interdependent DoG task. We would predict DoG in this implicit condition to be intermediate to an explicit promise and an explicit risk.

Another question for future studies is whether a promise or an explicit commitment was necessary. That is, whether the same effect would have been found on the basis of the interdependence conveyed in the description of the task alone. The findings of Koomen *et al*. [8] suggest that implicit commitments improve children’s delay of gratification. Thus, in the current study, we included promises to bolster both partners’ interdependence given the challenges of administering a cooperative marshmallow task online. Previous studies, however, suggest that children do not necessarily distinguish between different speech acts which signal social commitments, such as ‘I promise to do X’ and ‘I will do X’ [20], meaning children treat different cues (implicit or explicit) to social commitment similarly [31,32]. Future research might, therefore, wish to directly compare children’s DoG performance in response to implicit and explicit social commitments.

Although this study provides a validated new method for remote marshmallow paradigm testing, there were methodological challenges and limitations. First, owing to the online nature of the task, we could not predict the proportion of participants who would experience distractions in their home environments and subsequently be discounted from the reduced dataset, in which the testing conditions most closely mimicked those of a laboratory setting. To ensure our pre-registered sample size was met and in line with previous studies on children’s cooperation in interdependent decision-making contexts [29,33], additional data collection to bolster participant numbers in the reduced dataset was necessary. This forecasting difficulty may make this methodology unsuitable for time-sensitive projects. Our inclusion of a full and reduced dataset, however, enabled us to speak to the prevalence and effect that minor distractions can have on the results obtained, which might explain why remote experiments do not always neatly replicate in-laboratory analogues [25].

Second, to prevent participants from attempting to interact with a pre-recording, children were told that the confederate child could not hear nor see them but that they could hear and see him. This one-way communication likely prevented partners from establishing common ground, namely their mutual commitment to fulfilling their respective roles, which is thought to be a key principle of interdependence [6]. Though preschool children are keenly aware of what does and does not feature in one’s common ground [34–36], the fact that a condition difference was found for the duration and success of (younger) children’s DoG suggests that children understand the normative value of promises which, in this case, reduced the risk of the confederate eating his reward relative to when indecision was explicitly expressed.

The effects found in the present study reflect the behaviour of typically developing children living in northern England. To verify the extent to which the current findings are generalizable to all typically developing children, this paradigm ought to be replicated cross-culturally. Given the online nature of data collection, the value of the stoplight system for approximating the control of laboratory testing environments, and the fact that parents chose treats most relevant to their child’s preferences, this paradigm could readily be adapted for other linguistic and cultural settings. Moreover, this methodology, including the stoplight system and remote testing procedure, provides ample opportunity for cross-cultural comparisons exploring the ways in which interdependent DoG may be affected by social context.

From an applied perspective, promises have been found to promote cooperative behaviour in children, such that they are less likely to cheat [16] and more likely to tell the truth after promising [17,18]. Our finding that promises can increase the likelihood that children will delay gratification for cooperative purposes could also be harnessed to further enhance their cooperation. Further testing is needed in school environments in which a randomized controlled trial is feasible within a real-world DoG context in order to establish this method’s applied potential.

Delay of gratification has a rich history of study within individual contexts and outcomes. Here, we present a joint task in which outcomes were interdependent to explore this important skill in a cooperative context and find that children exert more effort to delay gratification when their partner promises to cooperate than when they express uncertainty about their willingness to wait. These findings are important not only because they demonstrate that delay of gratification studies can be conducted outside laboratory settings, but also because they illustrate the (de)motivating effect of explicit social commitments on delay of gratification for cooperative ends.

## Data Availability

The study was pre-registered. The pre-registration form is publicly accessible at: https://osf.io/ze9jb. Owing to ethical restrictions where no consent for public sharing was given, data and statistical scripts are available from the corresponding author or The University of Manchester’s Research Ethics Committee (research.ethics@manchester.ac.uk) upon request. Supplementary material is available online [37].
